# Supplementary material for: Successional Shifts in Soil C:N:P stoichiometry and its environmental controls during forest restoration in Huize County, Qujing, Yunnan Province
Source: PLoS One. 2026 Aug 21;21(8):e0356261. doi: 10.1371/journal.pone.0356261 (PMC13496410; doi:10.1371/journal.pone.0356261)
Supplement: Supplementary file 2 [file pone.0356261.s004.zip › Data and Code/Mantel/evergreen broadleaf forest/Mantel_heatmap_overall.pdf]

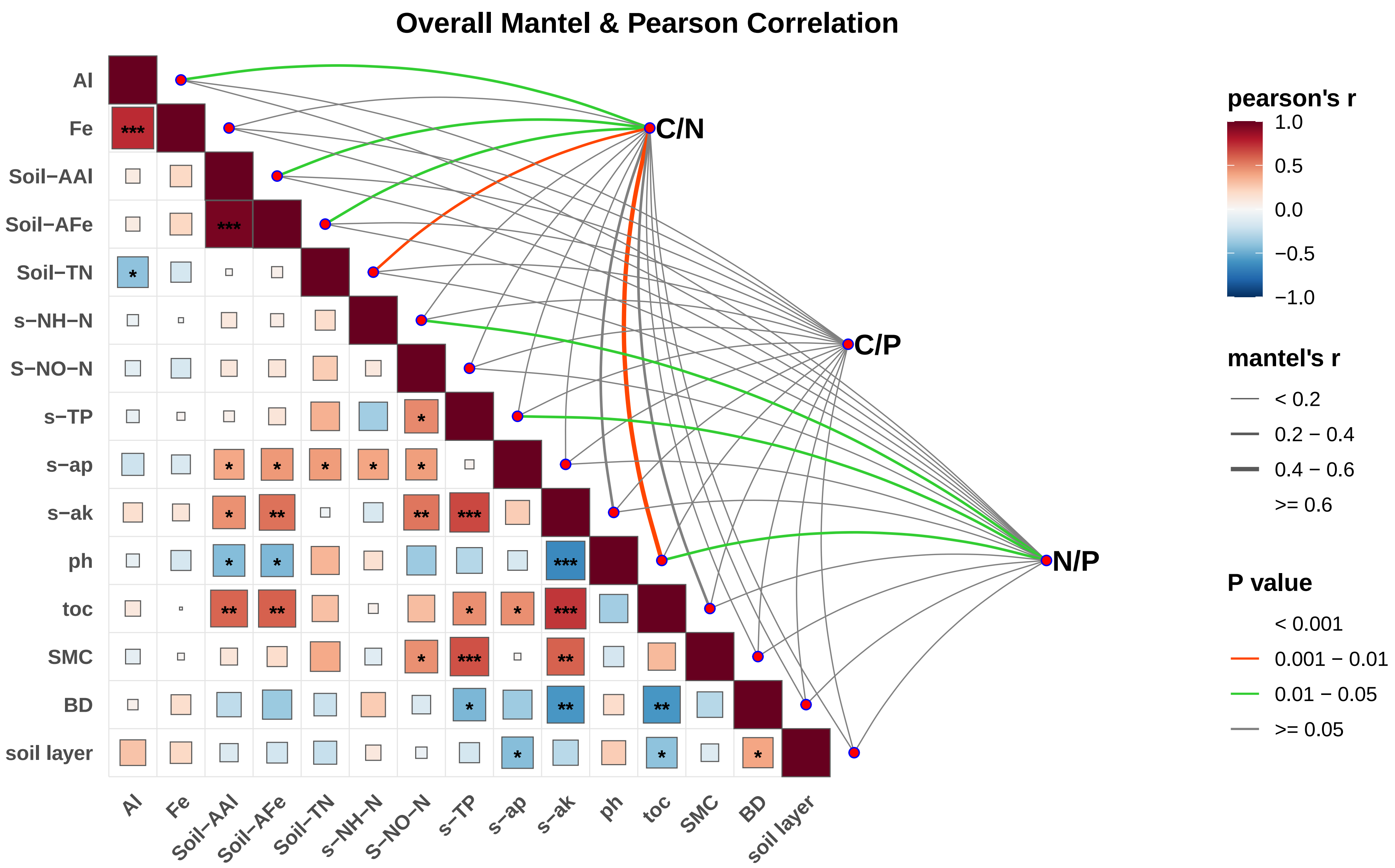

Overall Mantel & Pearson Correlation
Al
Fe
Soil−AAl
Soil−AFe
Soil−TN
s−NH−N
S−NO−N
s−TP
s−ap
s−ak
ph
toc
SMC
BD
soil layer
C/N
C/P
N/P
pearson's r
1.0
0.5
0.0
−0.5
−1.0
mantel's r
< 0.2
0.2 − 0.4
0.4 − 0.6
>= 0.6
P value
< 0.001
0.001 − 0.01
0.01 − 0.05
>= 0.05
